# Nitrogen deficiency restricts gall development by altering metabolic and hormonal networks in *Zizania latifolia*

**DOI:** 10.3389/fpls.2026.1754728

**Published:** 2026-05-07

**Authors:** Shenshen Zhang, Liwen Zheng, Jingtong Zhang, De-Ping Guo

**Affiliations:** Department of Horticulture, College of Agriculture and Biotechnology, Zhejiang University, Hangzhou, China

**Keywords:** *Zizania latifolia*, nitrogen deficiency, gall development, transcriptome, metabolome

## Abstract

*Zizania latifolia* is an important aquatic vegetable, and its edible gall is induced by the fungus *Ustilago esculenta*. Gall development is influenced by multiple factors. Nitrogen (N) is a central determinant of plant development and crop quality, yet how N modulates gall development in *Z. latifolia* remains largely unclear. Here, transcriptomic, metabolomic, and physiological analyses were integrated to investigate how N deficiency affects gall development at three stages: 7, 14, and 21 days after gall swelling. The present results showed that N deficiency reduced plant height, tiller number, and gall size, but promoted root length. There were decreases in photosynthetic performance, chlorophyll fluorescence parameters, and total nitrogen content with N deficiency. Concomitantly, gall tissues exhibited reduced levels of free amino acids and soluble proteins, alongside stage-dependent accumulation of soluble sugars. Multi-omics analyses revealed that N deficiency resulted in the coordinated suppression of N assimilation pathways, including GS/GOGAT, accompanied by enhanced lignin biosynthesis. In addition, N deficiency induced changes in redox-related processes, substantially altered phytohormone signaling pathways, and modified cell wall-related metabolic processes. Overall, the present study shows that N limitation disrupts carbon–nitrogen metabolic balance and source-sink allocation, thereby constraining gall expansion, structural development, and quality development. This work provides a physiological and molecular basis for optimizing N management in this crop.

## 1 Introduction

*Zizania latifolia* has been used as a vegetable in East Asia for hundreds of years (Xie et al., 2023). The edible part of the crop is its swollen stem (also called “gall”) induced by the smut fungus *Ustilago esculenta* at the seedling stage (Yan et al., 2018; Wu et al., 2024). Studies have demonstrated that gall formation is accompanied by pronounced host transcriptional and metabolic reprogramming, involving hormonal regulation, redox balance, and primary and secondary metabolism (Doehlemann et al., 2008; Skibbe et al., 2010; Wang et al., 2017). The gall formation of *Z. latifolia* represents a stem-derived storage organ formed under aquatic cultivation and is affected by numerous factors, such as light, temperature, phytohormones, and mineral nutrients (Yan et al., 2013; Ye et al., 2017; Li et al., 2019; Wang et al., 2021; Zhang et al., 2024). Accumulation of nutrients constitutes a fundamental physiological basis for stem enlargement and yield formation in *Z. latifolia* (Guo, 1988). However, studies on how mineral nutrition regulates growth and gall formation of *Z. latifolia* remain limited. Nitrogen (N) is a fundamental macronutrient that underpins nearly every aspect of plant growth and development. It is well known that N is a core component of amino acids, nucleic acids, chlorophyll, and numerous cofactors, and is indispensable for the synthesis of enzymes and structural proteins (de Bang et al., 2021; Francis et al., 2023). In plants, N availability strongly influences photosynthetic capacity, redox homeostasis, and primary and secondary metabolism, thereby shaping developmental trajectories and determining biomass accumulation (Tegeder and Masclaux-Daubresse, 2018; Chaput et al., 2020; Mu and Chen, 2021). Beyond its structural role, N is also a pivotal signaling molecule that integrates environmental cues with endogenous developmental programs through intricate transcriptional, post-transcriptional, and metabolic regulation (Forde, 2000; Orsel et al., 2002; Wang et al., 2018). Appropriate N fertilization can markedly promote the formation of productive tillers and increase gall yield (Xu et al., 2004). However, the mechanisms by which N affects gall development remain poorly understood.

In this study, hydroponic cultivation was used to investigate the responses of *Z. latifolia* growth and gall formation to N deficiency. By integrating physiological assays with transcriptomic and metabolomic analyses, we aimed to elucidate the mechanisms underlying N-mediated modulation of metabolic networks and developmental programs in this species. Our findings are expected to provide a fundamental physiological and molecular basis for understanding how N is involved in regulating gall development in *Z. latifolia*, which may help optimize N management strategies for the agricultural production of this crop.

## 2 Materials and methods

### 2.1 Plant materials and N treatments

*Zizania latifolia* cv. Chongjiao No.1 plants at the 6–8-leaf stage with uniform growth were transferred to hydroponic conditions at the Experimental Farm of Zhejiang University. Plants were maintained in tap water for two weeks prior to nutrient treatments. N treatments were initiated by replacing tap water with nutrient solutions (**Supplementary Table 1**). Two nitrogen regimes were applied: a control supplied with sufficient N (CK; 7.5 mM total N) and a N-deficient treatment without N (DN; 0 mM total N). In the CK treatment, N was supplied as 3.75 mM NH_4_NO_3_ to provide equimolar NH_4_^+^ and NO_3_^-^ (1:1), while all other macro- and micronutrients were supplied at identical concentrations in both treatments. During the experimental period, solution depth was maintained at approximately 25 cm by daily supplementation with deionized water, and continuous aeration was provided. Nutrient solutions were replaced every 1–2 weeks, and the initial pH of the solutions was adjusted to 6.5–7.0 at the beginning of each replacement cycle. The onset of gall swelling was defined as the first appearance of visible basal stem enlargement. The 7-day post-swelling stage was identified when the basal stem showed visible enlargement and 0.2 cm of gall tissue was visible from the middle region of the leaf sheath. Based on this criterion, the 14-day and 21-day post-swelling stages were defined 7 and 14 days later, respectively. Plants were monitored daily for stage determination. These stages (7, 14, and 21 days post-swelling, respectively) correspond to an early marketable stage, a post-market stage lacking commercial value, and a late overmature stage characterized by senescence. Plants were harvested at each stage. After the roots and foliage of plants were removed, galls were rinsed, sliced, frozen in liquid nitrogen, and stored at −80 °C for physiological, transcriptomic, and metabolomic analyses. Each treatment was performed with three biological replicates.

### 2.2 Photosynthetic performance and physiological traits

Gas exchange parameters and chlorophyll fluorescence traits, together with leaf chlorophyll content and root activity, were determined two weeks prior to the onset of stem swelling. Gas exchange parameters and chlorophyll fluorescence parameters were measured following the method described by Yan et al. (2013) with minor modifications. Gas exchange parameters were measured on fully expanded functional leaves. Net photosynthetic rate (Pn), stomatal conductance (gs), intercellular CO_2_ concentration (Ci), and transpiration rate (E) were recorded under a photosynthetic photon flux density of 1,000 μmol m^-^² s^-^¹ and an ambient CO_2_ concentration of approximately 400 μmol mol^-^¹ between 09:00 and 11:00 using a LI-6400XT portable photosynthesis system (LI-COR Biosciences, Lincoln, NE, USA). Chlorophyll fluorescence parameters were determined after dark adaptation for 30 min using a LI-600 portable fluorescence–stomatal conductance system (LI-COR Biosciences, Lincoln, NE, USA). The maximum quantum efficiency of PSII (F_v_/F_m_) and effective quantum yield of PSII (Φ_PSII_) were calculated following standard protocols. Leaf chlorophyll content was determined using the ethanol extraction method (Wang et al., 2022). Root activity was assessed using the triphenyl tetrazolium chloride (TTC) reduction method (Man et al., 2016).

### 2.3 Determination of gall physiological parameters and N-related enzyme activities

Gall morphological traits were evaluated by measuring gall length and maximum circumference, and the gall shape index was calculated as the ratio of length to maximum circumference. Total nitrogen content was determined by the Kjeldahl method (Singh et al., 2020). Soluble protein content was quantified using the Bradford assay (Bradford, 1976). Free amino acids, total sugars, soluble sugars, and lignin content were determined with commercial assay kits (Sangon Biotech Co., Ltd., Shanghai, China), following the manufacturer’s protocols. Crude fiber content was determined by the acid-alkali method (Hu et al., 2024). Phytohormone levels were quantified by high-performance liquid chromatography coupled with tandem mass spectrometry (HPLC-MS/MS) at ProNet Biotech Co., Ltd. (Nanjing, China). Enzyme activities of glutamine synthetase (GS), glutamate synthase (GOGAT), and glutamate dehydrogenase (GDH) were measured using assay kits (Sangon Biotech Co., Ltd., Shanghai, China). All measurements were conducted with three independent biological replicates. Unless otherwise specified, physiological parameters were determined on a fresh weight (FW) basis, whereas total nitrogen content was quantified on a dry weight (DW) basis.

### 2.4 RNA extraction, Illumina sequencing and transcriptomic data analysis

RNA extraction and sequencing were performed by Beijing Novogene Co., Ltd. (Beijing, China). A total amount of 1 μg RNA per sample was used for library preparation, with RNA integrity assessed using the Bioanalyzer 2100 system (Agilent Technologies, CA). After quality control, libraries were pooled based on concentration and target data size, prior to being subjected to Illumina sequencing. The target raw data (raw reads) were first processed using fastp software to obtain clean data (clean reads). Concurrently, Q20, Q30, and GC content of the clean data were also calculated. The reference genome and gene model annotation files were downloaded from the Genome Warehouse (GWH) database (accession number GWHBFHI00000000). Genome mapping was performed using HISAT2 v2.0.5. StringTie (v1.3.3b) was then used to assemble the mapped reads of each sample, and FeatureCounts v1.5.0-p3 was employed to count the number of reads mapped to each gene, after which FPKM values were calculated. Raw read counts generated by FeatureCounts were used as input for differential expression analysis. A differential expression analysis was performed between specified groups using the R software package DESeq2 (v1.20.0). The threshold for identifying significant differential expression was a corrected P-value of ≤ 0.05 and a |log_2_ (fold change) | of ≥ 1. Gene Ontology (GO) enrichment analysis and statistical enrichment in KEGG pathways (http://www.genome.jp/kegg/) of differentially expressed genes (DEGs) were implemented by the clusterProfiler R (v3.8.1) package, with a corrected P-value of less than 0.05.

### 2.5 Metabolite extraction and profiling analysis

Metabolite extraction, identification, and quantification were performed by Beijing Novogene Co., Ltd. (Beijing, China). Metabolites were extracted from 0.1 g of tissue samples ground in liquid nitrogen. UHPLC-MS/MS analyses were developed using a Vanquish UHPLC system (ThermoFisher, Germany) coupled to an Orbitrap Q Exactive™ HF mass spectrometer. Compound Discoverer 3.3 (CD3.3) was used to process the UHPLC-MS/MS data for peak alignment, metabolite extraction and quantification, with parameters set, and peak area corrected with the following parameters: peak areas were corrected using the first QC sample; mass tolerance was set to 5 ppm; signal intensity tolerance was set to 30%; and a minimum intensity threshold was applied. The mzCloud database was then integrated with the mzVault and MassList databases for matching and comparing peaks in order to facilitate metabolite identification and the subsequent quantification of relative levels. The annotation of metabolites was conducted using the KEGG database, HMDB database, and LIPIDMaps database. Principal components analysis (PCA) and Partial least squares discriminant analysis (PLS-DA) were performed using metaX. Student’s *t*-test was employed to calculate P values, and metabolites with VIP > 1, *p* < 0.05, and fold change ≥ 2 or FC ≤ 0.5 were defined as differential metabolites. The pairwise correlations between differential metabolites were calculated using Pearson’s method in R, and the resulting data were plotted using corrplot. Raw metabolite abundances obtained from LC-MS were log_2_-transformed and subsequently normalized by row (z-score scaling) to visualize relative accumulation patterns across samples. Six independent biological replicates were included per treatment group.

### 2.6 Correlation analysis

Correlation analysis was conducted using quantitative profiles of genes and metabolites. Pearson correlation coefficients were computed in R with the cor function. Within each pathway, gene–metabolite pairs with r > 0.80 and *p* < 0.05 were retained for network construction. These relationships were then visualized in Cytoscape (Shannon et al., 2003).

### 2.7 Real-time quantitative polymerase chain reaction analysis

The total RNA was isolated using the TRIzol method, and first-strand cDNA was synthesized using the *Evo M-MLV* RT Kit with gDNA Clean for qPCR (Accurate Biotechnology, China) according to the manufacturer’s instructions. Quantitative PCR was performed using the SYBR Green Premix *Pro Taq* HS qPCR Kit (Accurate Biotechnology, China) in a 96-well plate format. The relative expression levels were calculated according to the 2^-ΔΔCt^ algorithm. A total of three biological replicates were performed (*n* = 3). The complete set of primers utilized for RT-qPCR can be found in the supplementary dataset (**Supplementary Table 3**).

### 2.8 Statistical analysis

All statistical analyses were performed using GraphPad Prism (version 10.2.3). Data are presented as mean ± SD unless otherwise stated. For comparisons between two groups, statistical significance was evaluated using a two-tailed Student’s *t*-test. For experiments involving two independent factors, significance was assessed by two-way analysis of variance (ANOVA), followed by Tukey’s multiple-comparison test, *p* values < 0.05 were considered statistically significant. The number of independent biological replicates (*n*) is indicated in the figure legends.

## 3 Results

### 3.1 N deficiency suppresses plant growth

**Figure 1:**
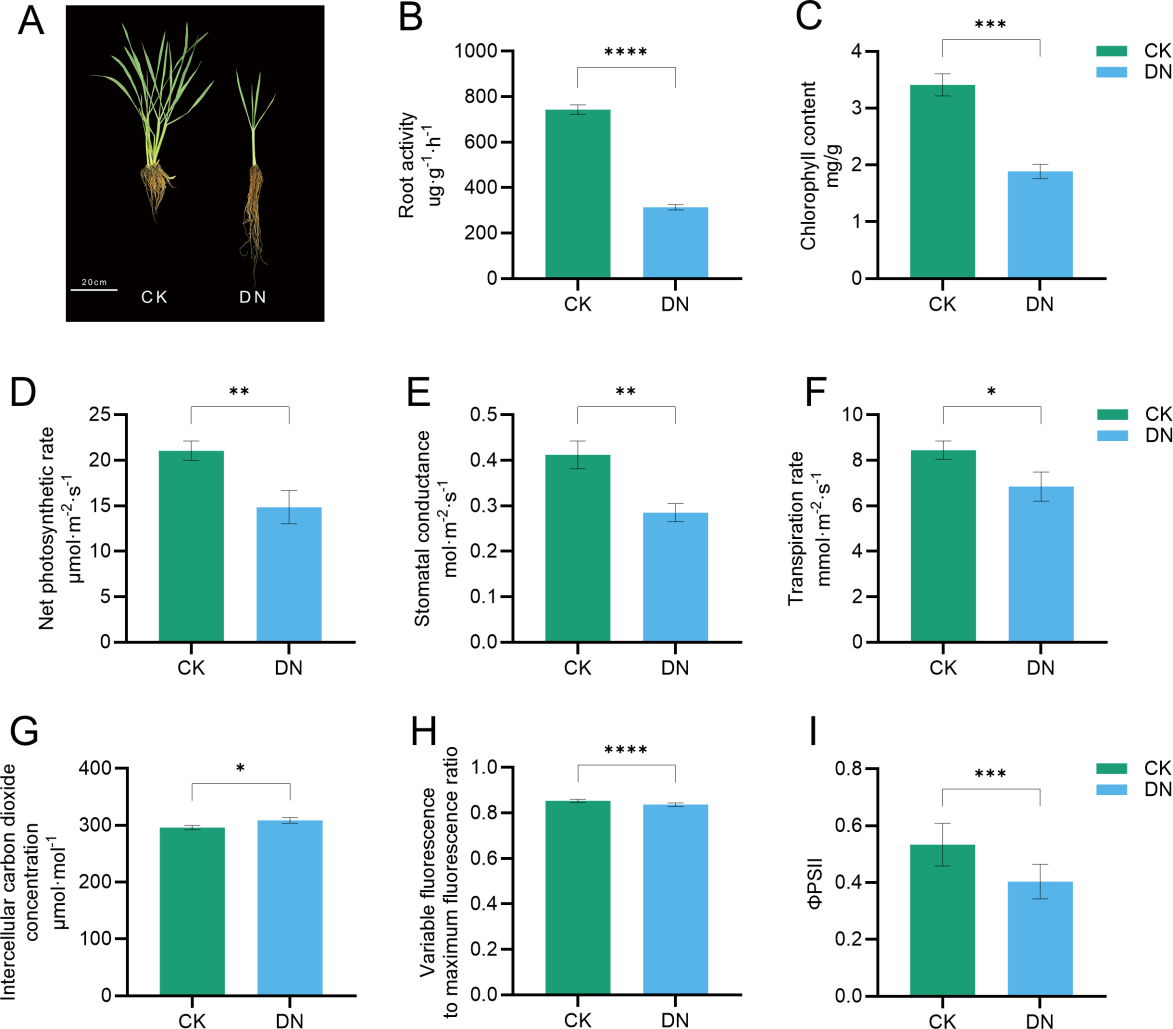
N deficiency impairs the growth and photosynthetic performance of *Z. latifolia.* **(A)** Representative images of whole plants grown under control (CK, 7.5 mM NH_4_NO_3_) and N-deficient (DN, 0 mM NH_4_NO_3_) conditions. **(B–I)** Measurements of root activity **(B)**, total chlorophyll content **(C)**, net photosynthetic rate (Pn) **(D)**, stomatal conductance (gs) **(E)**, transpiration rate **(E, F)**, intercellular carbon dioxide concentration (Ci) **(G)**, maximum quantum efficiency of PSII (F_v_/F_m_) **(H)**, and effective quantum yield of PSII (Φ_PSII_) **(I)** under CK and DN treatments. Data are presented as mean ± SD (*n* ≥ 3 biological replicates). *p < 0.05; **p < 0.01; ***p < 0.001; ****p < 0.0001. Scale bar = 20 cm.

Plants subjected to nitrogen-deficient treatment (DN; 0 mM total N) exhibited a marked reduction in plant height and tiller number, along with a great increase in root length compared to the control (CK; 7.5 mM total N) (**Figure 1A**). Meanwhile, root activity and chlorophyll content decreased significantly by 57.7% and 44.7%, respectively, under N deficiency (**Figures 1B, C**). Photosynthetic capacity was severely impaired, with net photosynthetic rate (Pn) declining by 29.5% (**Figure 1D**), accompanied by decreases in stomatal conductance (gs) and transpiration rate (E) by 30.8% and 18.9%, respectively, and a slight increase in intercellular carbon dioxide concentration (Ci) by 4.3% (**Figures 1E–G**). The maximal photochemical efficiency of PSII (F_v_/F_m_) and effective quantum yield of PSII (Φ_PSII_) were reduced by 2.0% and 24.4%, respectively (**Figures 1H, I**).

### 3.2 N deficiency alters morphological and biochemical traits of galls

**Figure 2:**
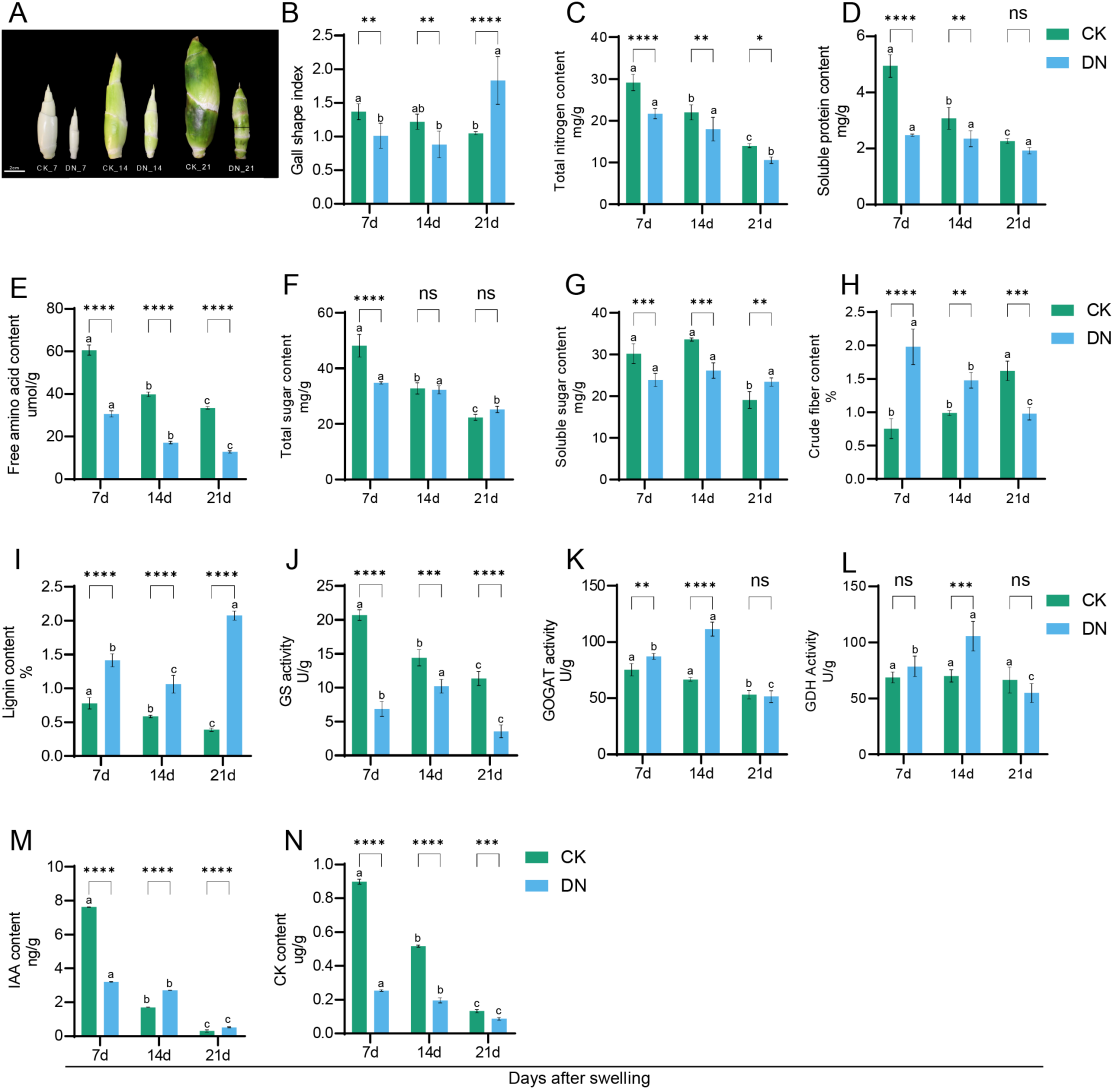
N deficiency affects biochemical and morphological changes in *Z. latifolia*. **(A)** Representative images of galls at 7, 14, and 21 days after swelling under control (CK) and N-deficient (DN) conditions. **(B–N)** Dynamic changes in morphological and biochemical traits during gall development, including gall shape index **(B)**, total nitrogen content **(C)**, soluble protein content **(D)**, free amino acid content **(E)**, total sugar content **(F)**, soluble sugar content **(G)**, crude fiber content **(H)**, lignin content **(I)**, glutamine synthetase (GS) activity **(J)**, glutamate synthase (GOGAT) activity **(K)**, glutamate dehydrogenase (GDH) activity **(L)**, indole-3-acetic acid (IAA) content **(M)**, and cytokinin (CK) content **(N)**. Data are presented as mean ± SD (*n* ≥ 3 biological replicates). *p < 0.05; **p < 0.01; ***p < 0.001; ****p < 0.0001. Scale bar = 2 cm. Different lowercase letters denote significant differences between time points within the same treatment (*p* ≤ 0.05); identical letters indicate no significant difference (*p* > 0.05).

N deficiency significantly reduced gall enlargement and altered its composition (**Figures 2A–I**). Morphological observations indicated that DN-treated galls were notably smaller, with reduced transverse growth and a more slender appearance (**Figures 2A, B**). Meanwhile, total nitrogen and soluble protein contents were significantly lower in DN plants across all stages (**Figures 2C, D**), and free amino acid levels in these plants declined by more than 60% at 21 days after swelling (**Figure 2E**). Carbohydrate metabolism also exhibited distinct changes. There was a significant reduction in total sugars at 7 days in DN plants (−27.8%), but not significantly different from CK at 21 days (+12.7%) (**Figure 2F**). Soluble sugar content decreased under DN at 7 and 14 days but increased at 21 days relative to CK, indicating a stage-dependent shift in soluble carbohydrate accumulation under N deficiency (**Figure 2G**). Crude fiber initially increased in DN plants (+162.1% at 7 days), then declined at 21 days (−39.7%) (**Figure 2H**), while lignin content steadily increased, exceeding fivefold that in CK by 21 days (**Figure 2I**). Furthermore, enzymes involved in N assimilation showed differential changes. Glutamine synthetase (GS) activity declined consistently in DN plants (**Figure 2J**). Glutamate synthase (GOGAT) activity increased at early stages but declined later (**Figure 2K**), and glutamate dehydrogenase (GDH) activity showed transient induction at 14 days (**Figure 2L**). Moreover, hormone contents were also affected by N deficiency. Indole-3-acetic acid (IAA) displayed a suppression at the early stage but elevation at the later stage (**Figure 2M**). In contrast, cytokinin (CK) levels were consistently lower in DN plants compared to CK plants across all stages (**Figure 2N**).

### 3.3 Transcriptomic analysis of genes during gall development under N deficiency

**Figure 3:**
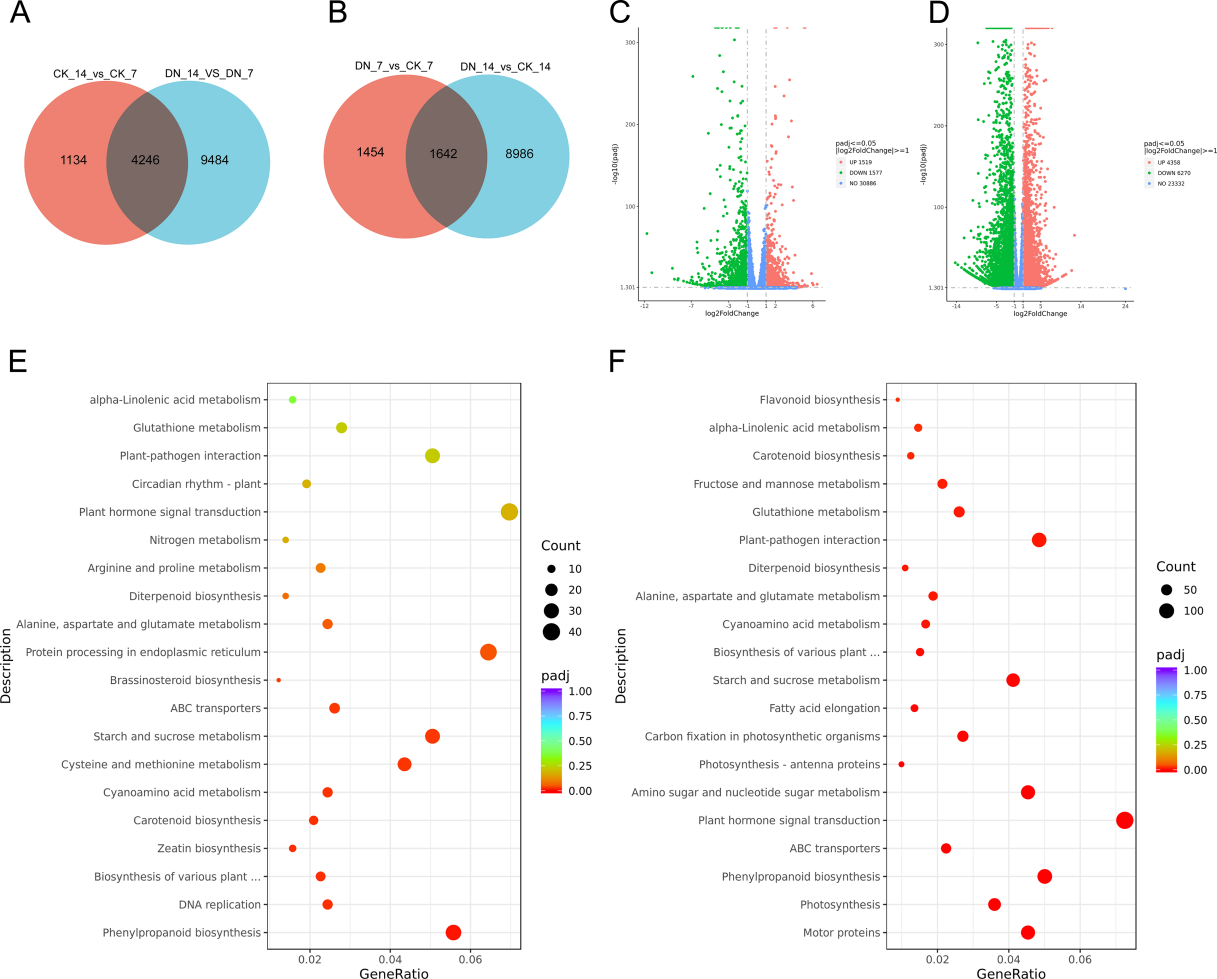
Transcriptomic landscape of N-deficient galls. **(A, B)** Venn diagrams showing DEGs for **(A)** CK_14_vs_CK_7 and DN_14_vs_DN_7, and **(B)** DN_7_vs_CK_7 and DN_14_vs_CK_14. **(C, D)** Volcano plots for **(C)** DN_7_vs_CK_7 and **(D)** DN_14_vs_CK_14 (red: up; green: down; blue: not significant; |log_2_FC| ≥ 1; FDR < 0.05). **(E, F)** KEGG enrichment for **(E)** DN_7_vs_CK_7 and **(F)** DN_14_vs_CK_14.

To explore the molecular mechanisms underlying gall development in *Z. latifolia* under N deficiency, transcriptomic analysis was conducted on samples grown under control (CK_7, CK_14) and N-deficient (DN_7, DN_14) conditions at two developmental stages (7 and 14 days). In our study, a total of 42.4 to 49.8 million reads were generated per sample, with mapping rates ranging from 88.75% to 92.44%. Of these, 37.9 to 44.5 million reads were uniquely mapped. The Q30 scores exceeded 94.83%, and GC content ranged from 51.85% to 53.13%, confirming high sequencing quality (**Supplementary Table 2**). Reads were mapped to the *Z. latifolia* reference genome, and the high unique mapping rate (>85%) indicated that the majority of transcripts were host-derived (**Supplementary Table 2**). Correlation analysis and principal component analysis (PCA) showed that biological replicates clustered tightly together and were clearly separated by treatment and developmental stage, indicating strong reproducibility and treatment-specific transcriptomic responses (**Supplementary Figures 2A, B**). In total, 14,864 differentially expressed genes (DEGs) were identified in CK_14_vs_CK_7 and DN_14_vs_DN_7, with 4,246 DEGs shared by the two comparisons. Under N deficiency, 12,082 DEGs were detected in DN_7_vs_CK_7 and DN_14_vs_CK_14, of which 1,642 were shared (**Figures 3A, B**). Volcano plots revealed that more genes were downregulated than upregulated during gall development. Specifically, 2,772 genes were downregulated and 2,608 were upregulated in CK_14_vs_CK_7; similarly, 7,898 genes were downregulated and 5,832 were upregulated in DN_14_vs_DN_7 (**Supplementary Figures 2C, D**). Notably, N deficiency triggered a substantially greater transcriptional regulation, with the DN_14_vs_DN_7 comparison showing the highest number of DEGs (13,730). Furthermore, the DN_14_vs_CK_14 comparison revealed more DEGs (10,628) than DN_7_vs_CK_7 (3,096), suggesting that N deficiency exerts a profound molecular impact as the gall develops (**Figures 3C, D**).

GO and KEGG enrichment analyses were performed on the DEGs. The top 30 significantly enriched GO terms were distributed across three major categories: biological processes (BP), cellular components (CC), and molecular functions (MF) (**Supplementary Figures 1A–D**). Upregulated DEGs were predominantly enriched in tetrapyrrole binding, heme binding, and photosynthesis-related terms, including photosynthesis, photosynthetic membrane, photosystem I/II, and photosystem I reaction center. Notably, these photosynthetic responses appeared decoupled from the developmental stages of the gall under N deficiency. By contrast, downregulated DEGs were enriched in terms related to carbohydrate metabolism and cell wall remodeling, such as hydrolase activity, glycosyl bond hydrolysis, and O-glycosyl compound metabolism (**Supplementary Figures 1E–H**), indicating synchronized repression of cell wall expansion and sugar turnover under stress or senescence. KEGG enrichment further identified 20 significantly enriched pathways (**Figures 3E, F**; **Supplementary Figures 2E, F**). Across all comparisons, core DEGs were commonly enriched in plant hormone signal transduction, plant–pathogen interaction, phenylpropanoid biosynthesis, ABC transporters, starch and sucrose metabolism, and glutamate metabolism. These enrichments also suggested stress/defense signaling and redox-associated processes as prominent components to respond to N deficiency.

Development-specific enrichments included glycolysis/gluconeogenesis, MAPK signaling, and the pentose phosphate pathway in CK_14_vs_CK_7 and DN_14_vs_DN_7, whereas stress-specific enrichments in DN_7_vs_CK_7 and DN_14_vs_CK_14 involved N metabolism, glutamate metabolism, and carotenoid biosynthesis, implying fundamental shifts in energy and N-flux coordination.

### 3.4 Integrated metabolomic and transcriptomic analysis of gall development under N deficiency

**Figure 4:**
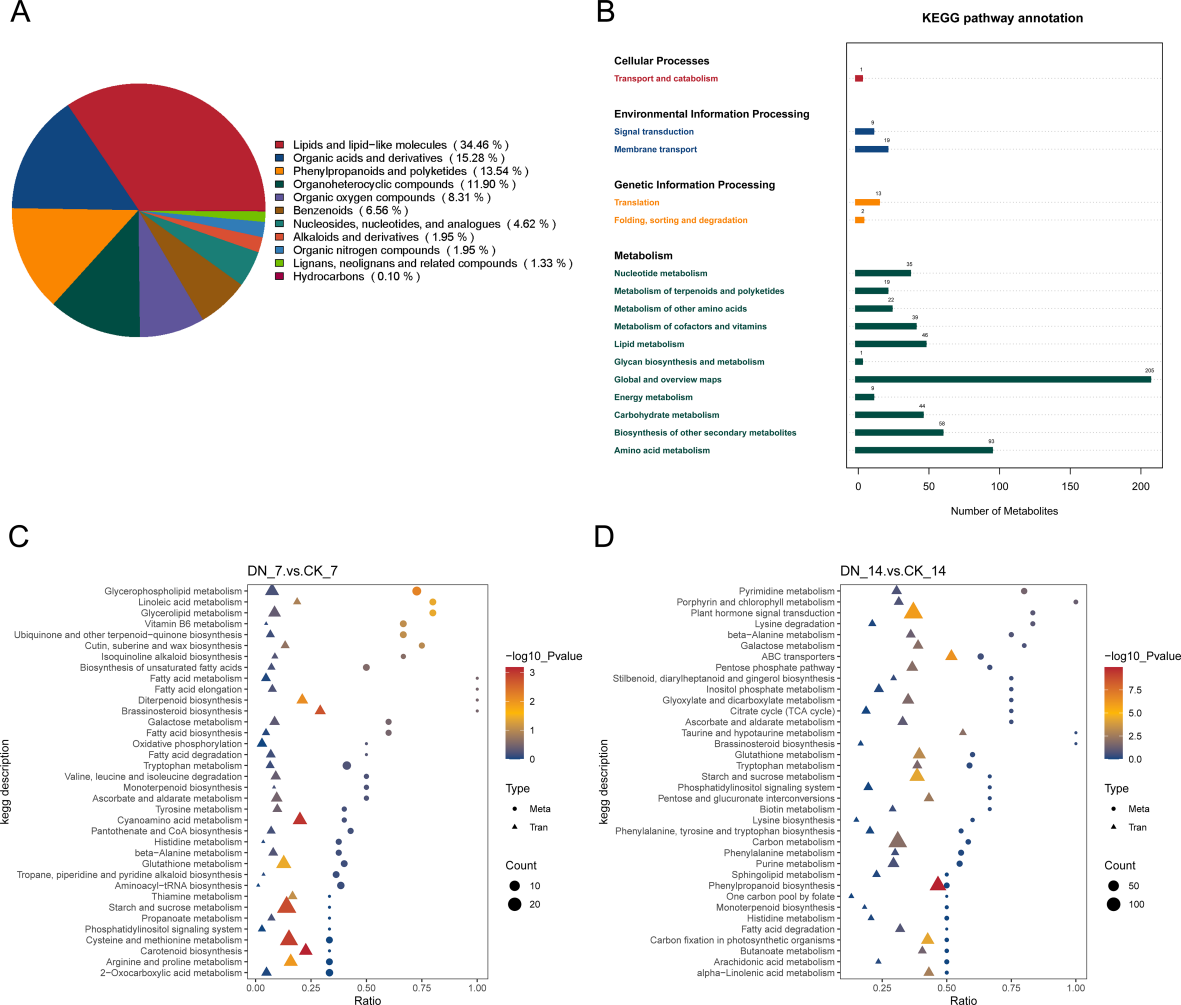
Multi-omics integration reveals metabolic hubs under N deficiency. **(A)** Composition of identified metabolites. **(B)** KEGG pathway annotation of identified metabolites. **(C, D)** Integrated pathway analysis for **(C)** DN_7_vs_CK_7, and **(D)** DN_14 vs CK_14.

To explore the metabolic responses underlying N deficiency–induced changes in gall development, we performed metabolomic profiling using UHPLC-MS/MS. There were a total of 1,327 metabolites identified. These included a wide range of compound classes, such as lipids and lipid-like molecules, phenylpropanoids and polyketides, organic acids and derivatives, organic oxygen compounds, organoheterocyclic compounds, benzenoids, nucleosides/nucleotides and analogues, lignans/neolignans and related compounds, organic nitrogen compounds, hydrocarbons, and others not classified within the above categories (**Figure 4A**). Across the developmental comparison groups, we identified 385 differentially accumulated metabolites (DAMs) in CK_14_vs_CK_7, including 208 upregulated and 177 downregulated DAMs. In DN_14_vs_DN_7, 651 DAMs were detected (403 upregulated, 248 downregulated). Comparison between N treatments revealed 478 DAMs in DN_7_vs_CK_7 (206 upregulated, 272 downregulated) and 615 DAMs in DN_14_vs_CK_14 (358 upregulated, 257 downregulated) (**Supplementary Figures 3A–D**). KEGG pathway enrichment of DAMs across all groups revealed major metabolic shifts in amino acid metabolism, secondary metabolite biosynthesis, carbohydrate metabolism, and lipid metabolism (**Figure 4B**). Notably, tryptophan metabolism and brassinosteroid biosynthesis were consistently enriched in both N treatment comparisons (DN_7_vs_CK_7 and DN_14_vs_CK_14), whereas phenylpropanoid biosynthesis, alanine/aspartate/glutamate metabolism, nicotinate and nicotinamide metabolism, and glycerophospholipid metabolism were co-enriched in both comparisons between developmental stages (CK_14_vs_CK_7 and DN_14_vs_DN_7) (**Supplementary Figures 3E–H**).

To further decipher the molecular basis by which N deficiency modulates gall development, we integrated DAMs with DEGs. Transcriptome and metabolome co-enrichment analyses revealed consistent pathway-level changes, particularly in starch and sucrose metabolism, phenylpropanoid biosynthesis, plant hormone signal transduction, and tryptophan-related pathways (**Figures 4C, D**; **Supplementary Figures 4A, B**). These pathways are central to hormonal signaling, structural remodeling, and metabolic reprogramming during gall development. Global metabolic mapping using iPath showed that differential signals from all four comparisons (CK_14_vs_CK_7, DN_14_vs_DN_7, DN_7_vs_CK_7, and DN_14_vs_CK_14) were widely distributed across the metabolic network (**Supplementary Figures 4C–F**), indicating extensive metabolic changes. Development-related comparisons were mainly associated with core metabolic processes such as carbohydrate and amino acid metabolism, whereas N deficiency additionally affected lipid and secondary metabolism.

### 3.5 N deficiency affects N transport and assimilation

**Figure 5:**
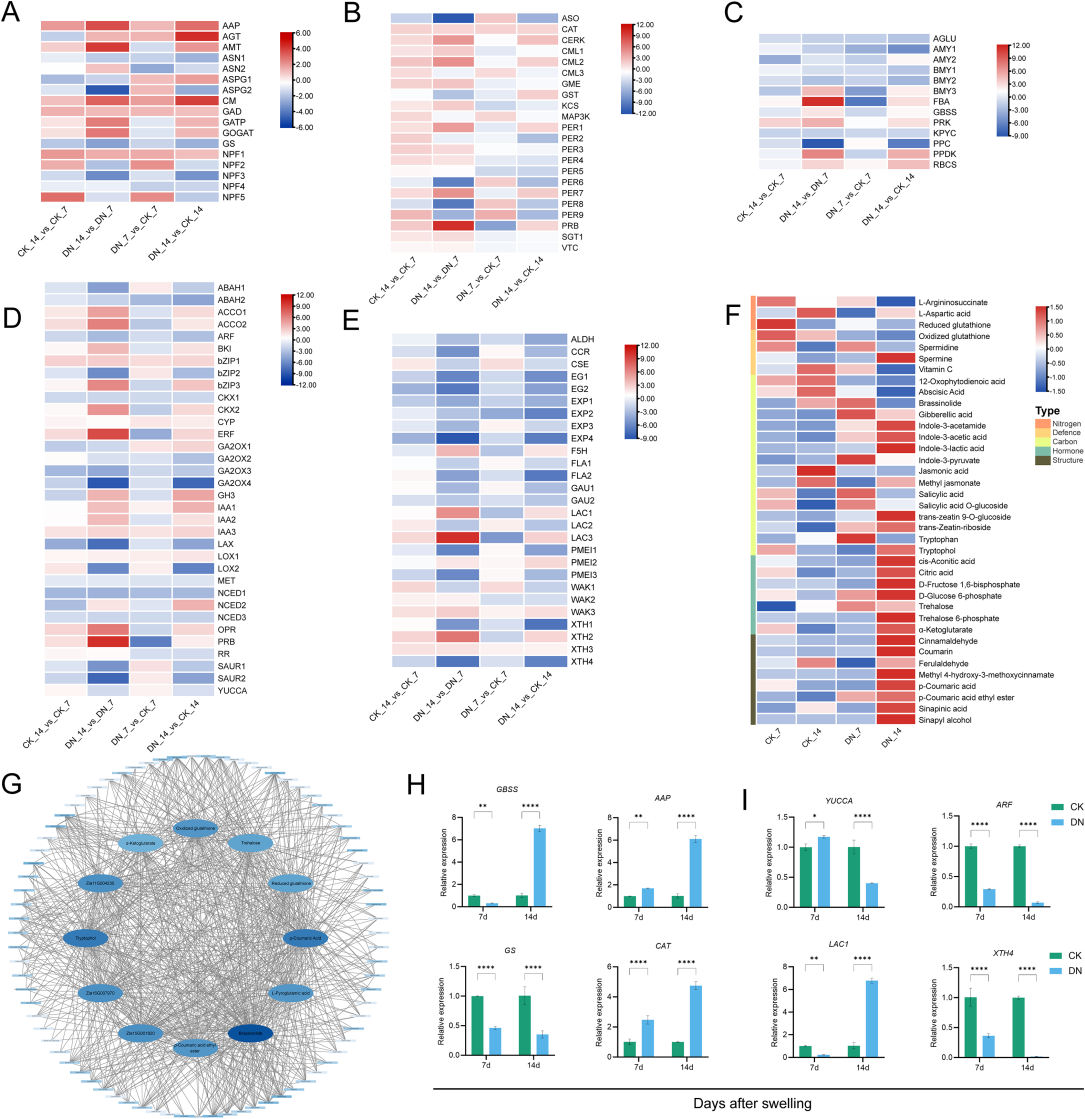
Integrated primary metabolism and signaling pathways linking DEGs and DAMs under N deficiency. **(A–F)** Pathway maps integrating DEGs and DAMs for **(A)** nitrogen metabolism, **(B)** redox homeostasis and pathogen-associated responses, **(C)** Carbon metabolism, **(D)** Hormone biosynthesis and signal transduction, **(E)** Phenylpropanoid metabolism and cell wall remodeling (red: up; blue: down; |log_2_FC|≥1; FDR<0.05), **(F)** Relative metabolite abundance of primary DAMs across the four sample groups (CK_7, CK_14, DN_7, and DN_14). **(G)** Correlation network between DEGs and DAMs. **(H, I)** RT-qPCR analysis of the relative expression of eight representative genes across the four groups (CK_7, DN_7, CK_14, and DN_14). Data are presented as mean ± SD (*n* ≥ 3 biological replicates). *p < 0.05; **p < 0.01; ***p < 0.001; ****p < 0.0001.

To elucidate N utilization strategies during gall development under N deficiency, we analyzed 17 genes involved in N uptake, assimilation, and redistribution, along with associated N-related metabolites (**Figures 5A, F**). Our results indicated that, at the transporter level, amino acid permease (*AAP*) and nitrate transporter (*NPF1*) were consistently upregulated under DN treatment across both developmental stages. In contrast, *NPF3* and *NPF4* were suppressed in DN plants, whereas *NPF2* and *NPF5* exhibited a biphasic response, being transiently upregulated at DN_7 but markedly downregulated at DN_14. Interestingly, the ammonium transporter (*AMT*) was downregulated at DN_7 but significantly upregulated at DN_14, suggesting a delayed adaptation in ammonium uptake.

Key genes in the N assimilation pathway were generally suppressed under DN conditions. Glutamine synthetase (*GS*) and asparagine synthetase (*ASN1/2*) were downregulated throughout development, indicating reduced N assimilation capacity. Glutamate synthase (*GOGAT*) was repressed at DN_7, but upregulated at DN_14 (**Figure 5A**), possibly reflecting a compensatory recovery of glutamate biosynthesis.

In nitrogen recycling, L-asparaginase (*ASPG1*) was consistently induced under DN conditions, while *ASPG2* showed transient upregulation at DN_7 and was repressed at DN_14. Notably, chorismate mutase (*CM*) was strongly induced under DN, with expression increasing from a 3.5-fold change at DN_7 to 15.0-fold at DN_14 (**Figure 5A**), suggesting a shift in chorismate allocation and possible changes in aromatic amino acid metabolism under N deficiency. Additionally, transcriptional regulation of the γ-aminobutyric acid (GABA) shunt was found (**Figure 5A**). Glutamate decarboxylase (*GAD*) was strongly induced under DN, while GABA transaminase (*GATP*) showed a temporal shift, being suppressed at DN_7 and upregulated at DN_14. Alanine-glyoxylate aminotransferase (*AGT*) was progressively induced, with a fold change rising from 2.4 to 19.6, suggesting enhanced aminotransferase activity and a possible adjustment of carbon–nitrogen metabolic flux under N deficiency.

These coordinated transcriptional changes were accompanied by notable shifts in related metabolites (**Figure 5F**). Levels of reduced glutathione, a key redox-related metabolite, and L-argininosuccinate, an intermediate in arginine biosynthesis, were high in CK_7 but markedly decreased in DN_14, suggesting impaired metabolic homeostasis under prolonged N deficiency. Meanwhile, L-aspartic acid, a precursor for asparagine and nucleotide biosynthesis, was sharply reduced in DN_7, which was in line with the reduced expression of *ASNs*.

### 3.6 N deficiency alters stress responses and defense signaling during gall development

To evaluate stress- and defense-associated responses with N deficiency during gall development, we examined DEGs mapped to enriched KEGG pathways, including MAPK signaling, plant–pathogen interaction, and glutathione metabolism, and further inspected representative antioxidant-related genes together with relevant metabolites (**Figures 5B, F**). In the antioxidant defense system, L-ascorbate oxidase (*ASO*) was upregulated at DN_7 but downregulated at DN_14, while catalase (*CAT*) was consistently induced under DN treatment, indicating dynamic modulation of redox-related defenses under N deficiency. By contrast, genes involved in ascorbate biosynthesis, including guanosine diphosphate (GDP)-mannose 3, 5-epimerase (*GME*) and GDP-L-galactose phosphorylase (*VTC*), were repressed under N deficiency. Meanwhile, glutathione metabolism showed clear transcriptional and metabolic changes. Glutathione S-transferase (*GST*) was upregulated at DN_7 and downregulated at DN_14, corresponding to a sharp reduction in oxidized glutathione levels at DN_14. Similarly, reduced glutathione was depleted in DN plants, indicating disturbed glutathione homeostasis and a weakened antioxidant buffering capacity.

The mitogen-activated protein kinase (MAPK) signaling cascade exhibited a stage-specific response (**Figure 5B**). Calcium-binding protein (*CML3*) and mitogen-activated protein kinase kinase kinase (*MAP3K*) were induced at DN_7, but downregulated at DN_14, while *CML2* was suppressed across gall development, indicating an early activation and subsequent attenuation of stress-responsive signaling.

In the plant–pathogen interaction pathway, chitin elicitor receptor kinase (*CERK*), 3-ketoacyl-CoA synthase (*KCS*), suppressor of G2 allele of *SKP* (*SGT1*), and pathogenesis-related protein (*PRB*) were downregulated at DN_7, with partial recovery of *CERK* and *PRB* expression at DN_14 (**Figure 5B**). This pattern suggests a transient suppression of defense-related signaling under N stress, possibly reflecting resource allocation trade-offs. Peroxidases (*PERs*), which participate in cell wall remodeling and redox homeostasis, exhibited divergent expression profiles. For instance, *PER1* was downregulated at DN_7, but upregulated at DN_14, while *PER2* was progressively repressed, showing increasing suppression during gall development.

Metabolite profiling further supported these transcriptional changes (**Figure 5F**). Vitamin C (ascorbate) accumulated at CK_14, but sharply declined at DN_14, reflecting impaired ROS buffering capacity under stress. Meanwhile, oxidized glutathione also decreased at DN_14, further indicating disruption of glutathione-related redox balance during the late stage. Spermidine and spermine showed stage-dependent changes under N deficiency, indicating that polyamine-related stress responses varied across gall development.

### 3.7 N deficiency changes carbon allocation during gall development

To investigate how N deficiency affects carbon allocation during gall development, we analyzed 13 genes involved in photosynthesis, glycolysis, starch degradation, fermentation, and trehalose metabolism, alongside relevant differentially accumulated metabolites (**Figures 5C, F**). In the photosynthesis-related pathway, both the phosphoribulokinase (*PRK*) gene and the ribulose bisphosphate carboxylase small subunit (*RBCS*) gene were upregulated in DN plants, indicating a potential compensatory adjustment in carbon assimilation under N limitation. Meanwhile, glycolytic activity appeared partially repressed, with a downregulated pyruvate kinase (*KPYC*) gene under DN. Fructose-bisphosphate aldolase (*FBA*), and pyruvate phosphate dikinase (*PPDK*) were downregulated at DN_7 but showed partial recovery at DN_14. These changes were supported by the accumulation of D-fructose 1, 6-bisphosphate and D-glucose 6-phosphate in DN_14, suggesting increased glycolytic intermediate retention and delayed carbon catabolism under N stress.

Phosphoenolpyruvate carboxylase (*PPC*), a key component of anaplerotic CO_2_ fixation, was upregulated at DN_7, but suppressed at DN_14, indicating dynamic flux modulation between glycolysis and the tricarboxylic acid (TCA) cycle. This was accompanied by the accumulation of citric acid, cis-aconitic acid, and α-ketoglutarate at DN_14, which may indicate altered TCA-cycle turnover during the late stage of gall development under N deficiency.

In the starch- and trehalose-related carbohydrate metabolism module, α-amylase (*AMY1)*, β-amylases (*BMY1/2*), and α-glucosidase (*AGLU*) genes were consistently downregulated in DN plants, whereas *AMY2*, *BMY3*, and granule-bound starch synthase (*GBSS*) were suppressed at DN_7 but recovered at DN_14. These transcriptional changes were accompanied by transient increases in trehalose and trehalose 6-phosphate at DN_7, followed by declines at DN_14, suggesting an early adjustment of trehalose-related carbohydrate metabolism under N deficiency.

### 3.8 N deficiency reshapes phytohormone-mediated gall development

To elucidate how phytohormones influence developmental plasticity under N deficiency, we analyzed the expression of 34 genes involved in major hormone biosynthesis and signaling pathways, as well as hormone-related metabolites (**Figures 5D, F**). In the auxin pathway, genes of key transporters and transcriptional regulators were differentially expressed. Auxin response factor (*ARF*) and auxin transporter-like protein (*LAX*) genes were suppressed under DN, while auxin-responsive gene family members (*IAA1/3*) were upregulated. Notably, indole-3-pyruvate monooxygenase (*YUCCA*) and small auxin-up RNA (*SAUR1/2*) were induced at DN_7 but repressed at DN_14, consistent with an early enhancement of auxin-related responses. In contrast, indole-3-acetic acid-amido synthetase (*GH3*) and *IAA2* were downregulated at DN_7, but upregulated at DN_14, suggesting stage-specific modulation of auxin conjugation and homeostasis. Metabolite profiling also supported these trends (**Figure 5F**). The results indicated that although indole-3-pyruvate (IPA) was upregulated relative to CK, its levels declined from DN_7 to DN_14, mirroring changes in tryptophan, which increased initially and then decreased markedly in DN plants. This pattern is consistent with the repression of *YUCCA* at DN_14, and suggests that auxin precursor accumulation was enhanced at the early stage, whereas later auxin biosynthesis may have been constrained by reduced tryptophan availability. Additional auxin-related metabolites such as indole-3-lactic acid and indole-3-acetamide were also elevated at DN_14, indicating a shift in auxin-related metabolism during the later phase of gall development. Moreover, tryptophol accumulated to its highest level at DN_14, suggesting that N deficiency may have altered the partitioning of tryptophan-derived indole metabolites, potentially affecting auxin-related metabolic homeostasis. This trend is consistent with the strong induction of *GH3* at DN_14 and suggests a shift from early activation of auxin synthesis toward a later auxin homeostatic state involving conjugation and redistribution of indole-related metabolites (**Figures 5D, F**).

In the cytokinin (CK) pathway, cytokinin dehydrogenase (*CKX1*) and two-component response regulator (*RR*) genes were suppressed under DN, while *CKX2* showed a recovery at DN_14. These changes coincided with increased levels of trans-Zeatin-riboside and trans-Zeatin 9-O-glucoside at DN_14, suggesting an adjustment of cytokinin homeostasis.

In the gibberellin (GA) pathway, the gibberellin 2-beta-dioxygenase (*GA2OX1*) gene was induced, while *GA2OX2/3/4* genes were repressed under DN. These changes coincided with elevated gibberellic acid at DN_7, suggesting altered GA homeostasis under N limitation.

For brassinosteroid (BR) signaling, brassinosteroid insensitive kinase inhibitor (*BKI*) was downregulated at DN_7, but upregulated at DN_14, while cytochrome P450 family (*CYP*) was consistently induced. These transcriptional changes were correlated with elevated brassinolide in DN_7, indicating an early burst of BR signaling potentially contributing to N stress adaptation.

In the abscisic acid (ABA) pathway, the bZIP transcription factor (*bZIP1*) was upregulated under DN, whereas the ABA catabolic gene abscisic acid 8’-hydroxylase *(ABAH2)* and the biosynthetic genes 9-cis-epoxycarotenoid dioxygenase (*NCED1/3*) were repressed. *ABAH1* and *bZIP2* were induced at DN_7 but declined at DN_14, whereas *NCED2* and *bZIP3* exhibited opposite trends. These results indicate stage-specific modulation of ABA biosynthesis, catabolism, and signaling under N deficiency. Consistent with the repression of key biosynthetic genes, ABA content was markedly reduced under DN, with a further decline from DN_7 to DN_14.

Both 1-aminocyclopropane-1-carboxylate oxidase (*ACCO1/2*) and ethylene-response factor (*ERF*) genes, which are involved in the ethylene (ET) pathway, were repressed at DN_7 and recovered at DN_14. This was consistent with the accumulation of methionine, an ethylene precursor, at DN_14, possibly reflecting reactivation of ET biosynthesis.

Jasmonic acid (JA) signaling showed a biphasic pattern similar to that of ET. The lipoxygenase (*LOX1*) gene was consistently upregulated, while the *LOX2* gene was transiently induced at DN_7. However, 12-oxophytodienoate reductase (*OPR*) was downregulated early but recovered at DN_14. Meanwhile, methyl jasmonate, jasmonic acid, and 12-oxophytodienoic acid were elevated in CK_14, but sharply declined at DN_14, suggesting weakened jasmonate accumulation under prolonged N deficiency.

In the salicylic acid (SA) pathway, pathogenesis-related protein (*PRB*) overlapped with defense regulation (see plant–pathogen interactions), and salicylic acid and salicylic acid O-glucoside accumulated at DN_7 and then declined at DN_14 (**Figure 5F**), reflecting stage-specific defense signaling.

Additionally, epigenetic modulation may also contribute to hormonal plasticity. The DNA (cytosine-5)-methyltransferase (*MET*) gene was repressed under DN, implying a possible contribution of DNA methylation-related regulation to hormonal plasticity under N stress (**Figure 5D**).

### 3.9 N deficiency modulates phenylpropanoid metabolism and cell wall remodeling during gall development

N deficiency was also affected the structural characteristics of gall tissues through the regulation of 27 genes involved in phenylpropanoid metabolism and cell wall dynamics, along with related metabolite profiles (**Figures 5E, F**). In the lignin biosynthesis and phenylpropanoid pathway, several key genes showed dynamic expression changes under DN conditions. Cinnamoyl-CoA reductase (*CCR*), caffeoylshikimate esterase (*CSE*), and laccase (*LAC2*) genes were significantly upregulated at DN_7, but suppressed at DN_14, consistent with the transient activation of lignin precursor biosynthesis and early lignification-related processes. In contrast, ferulate 5-hydroxylase (*F5H*), *LAC1*, and *LAC3* genes were downregulated at DN_7 and upregulated at DN_14, indicating a delayed induction of lignin polymerization-related enzymes. Aldehyde dehydrogenase (*ALDH*) genes were consistently repressed under N deficiency. These transcriptional shifts were partly reflected in metabolite accumulation patterns. Ferulaldehyde markedly declined at DN_7, whereas sinapyl alcohol accumulated at DN_14, and this temporal divergence aligns with the expression dynamics of *F5H* and *LAC1*. In addition, cinnamaldehyde, coumarin, and *p*-coumaric acid ethyl ester, phenylpropanoid-derived compounds associated with lignification and defense, were significantly enriched at DN_14, suggesting activation of cell wall reinforcement and stress-responsive secondary metabolism during the late stage of gall development.

Genes involved in cell wall modification, including wall-associated receptor kinase (*WAK3*), xyloglucan endotransglucosylase/hydrolase (*XTH3*), and pectinesterase inhibitor (*PMEI2*), were consistently induced under DN conditions, indicating enhanced signaling and crosslinking activity in the wall matrix. In contrast, expression of genes such as fasciclin-like arabinogalactan protein (*FLA2*), *WAK2*, expansins (*EXP1/2/4*), endoglucanase (*EG1/2*), *XTH1/4*, and galacturonosyltransferase (*GAU2*) was generally downregulated, suggesting reduced wall loosening and biosynthesis. A subset of wall-modifying genes, *FLA1*, *WAK1*, *EXP3*, *PMEI1/3*, and *GAU1*, were induced at DN_7, but suppressed at DN_14, revealing early but unsustained remodeling activity. Consistently, several wall-related metabolites showed differential accumulation. Sinapinic acid, *p*-coumaric acid, and methyl 4-hydroxy-3-methoxycinnamate were elevated at DN_14, supporting enhanced accumulation of phenylpropanoid-related metabolites.

### 3.10 Metabolic network and relative expression of DEG genes

To further dissect coordinated regulatory relationships among the key pathways identified above, we performed a correlation analysis between DEGs and DAMs and constructed a gene–metabolite association network, highlighting 12 nodes (genes and metabolites) with the highest correlation coefficients (**Figure 5G**). This analysis extended our previous findings and highlighted several genes with strong correlation coefficients, including *AAP* (*Zla11G004230*) and *XTH3/4* (*Zla15G001820*, *Zla16G007970*), as well as multiple metabolites showing strong associations with these genes. Obviously, hormone-related metabolites such as brassinolide and tryptophol, redox-associated compounds including oxidized glutathione and reduced glutathione, and carbon metabolism intermediates such as α-ketoglutarate, trehalose, and L-pyroglutamic acid emerged as key nodes in the network. Additionally, phenylpropanoid pathway intermediates like *p*-coumaric acid and *p*-coumaric acid ethyl ester also showed strong gene–metabolite associations. Furthermore, eight representative DEGs were selected for relative expression analysis across CK_7, DN_7, CK_14, and DN_14. RT-qPCR validation confirmed that the expression trends were consistent with those obtained from the RNA-seq analysis (**Figures 5H, I**).

## 4 Discussion

### 4.1 N deficiency influences plant growth and carbon-nitrogen metabolic balance

N availability is essential for plant growth and development, particularly for crops with high economic value (Govindasamy et al., 2023). In this study, our results indicated that N deficiency significantly inhibited vegetative growth, gall development, and photosynthetic activity of *Z. latifolia*, but promoted root elongation (**Figure 1**, **2**). It is generally recognized that root elongation as a typical response to limited N availability possibly reflects a compensatory adaptation to enhance N acquisition from the rhizosphere (Kant et al., 2011; Lopez et al., 2023). Previous studies have shown that inhibitory effects of N deficiency on photosynthesis can be attributed to both metabolic and anatomical constraints (Mu and Chen, 2021). In our study, decreases in chlorophyll content and net photosynthetic rate (Pn) under N deficiency (**Figure 1D**) were observed; this may be associated with impaired N-dependent assembly of photosynthetic complexes and reduced energy conversion efficiency (Lawson and Blatt, 2014; Mu and Chen, 2021). Consistent with this observation, declines in gs and E together with a slight rise in Ci indicate that the reduction in CO_2_ assimilation was driven primarily by nonstomatal factors rather than by stomatal closure (**Figures 1E–G**) (Mu and Chen, 2021). The relatively small change in F_v_/F_m_ suggests that the maximal photochemical efficiency of PSII was only modestly affected under N deficiency. By contrast, the marked decline in Φ_PSII_ reflects a pronounced limitation in the utilization of photochemical energy during downstream carbon assimilation under N deficiency (**Figures 1H, I**) (Baker, 2008). N deficiency also provokes systemic signaling changes involved in ABA and ROS metabolism that modulate stomatal behavior and stress responses (Tewari et al., 2007). In our study, however, these signals appear to have contributed mainly to acclimatory adjustments rather than the immediate suppression of photosynthetic capacity, consistent with previous reports (Tewari et al., 2007; Mu et al., 2018). In the present study, photosynthetic capacity was suppressed under N-deficient conditions (**Figure 1**), indicating that N limitation may override or constrain the photosynthetic enhancement sometimes associated with biotrophic interactions. Specifically, it has been observed that photosynthesis increases in source leaves of *Ustilago maydis*-infected maize and *U. esculenta*-infected *Z. latifolia*, suggesting that biotrophic colonization can enhance source capacity to support gall development (Horst et al., 2010; Yan et al., 2013; Cheaib and Killiny, 2025). This reduction in source capacity by N deficiency is likely to limit carbon supply to the developing gall and thereby contribute to restricted gall expansion (Burnett et al., 2018; Cheaib and Killiny, 2025).

N and carbon (C) metabolism are tightly interconnected in plants, and their coordinated regulation is essential for growth and organ development (Zheng, 2009). Under N-deficient conditions, plants typically engage adaptive programs that reconfigure N acquisition and assimilation and rebalance C/N metabolism at both transcriptional and metabolic levels (Masclaux-Daubresse et al., 2010). In this study, the expression of several key genes involved in N uptake and assimilation was downregulated under N deficiency, which likely constrained the GS/GOGAT cycle and thereby limited amino acid biosynthesis (**Figure 5A**) (Fortunato et al., 2023). These molecular responses were reflected by the observed reductions in total nitrogen content, free amino acids, and soluble proteins under N deficiency (**Figure 2**). Notably, GS activity displayed a partial recovery at later stages during prolonged N limitation, suggesting that acclimatory regulation of ammonium assimilation may occur during gall maturation (**Figure 2J**) (Masclaux-Daubresse et al., 2010). On the carbon side, N deficiency triggered large-scale reconfiguration of carbon metabolism, possibly to redirect energy and carbon skeletons in response to limited N availability (Ke et al., 2024). It has been reported that α-ketoglutarate (2-OG) serves as a central signal in N metabolism and a crucial metabolic bridge for ammonium assimilation and C/N coupling (Zhang et al., 2018). Glucose-6-phosphate (G6P) operates at a metabolic crossroads by linking glycolysis and trehalose metabolism (Chaput et al., 2020), thereby integrating carbon flux partitioning with cellular energy and redox demands. Trehalose-6-phosphate (T6P) acts as a stress protectant or storage sugar, and a key regulator of carbon allocation in plants (Schluepmann et al., 2003). In addition, fructose-1,6-bisphosphate aldolase (FBA) participates in central carbon flux through glycolysis and the Calvin cycle (Streb and Zeeman, 2012). Against this metabolic backdrop, starch synthesis and turnover provide a direct readout of how plants adjust carbon storage and remobilization during gall development. In rice, *OsGBSS* is a major gene controlling amylose synthesis and directly affects starch composition in the endosperm and pollen (Tian et al., 2009). *Atbam3* mutants exhibit excessive starch accumulation in leaves, while *OsBAM2* is implicated in transient starch degradation within plastids (Sugimura et al., 2023), supporting a role for β-amylases in regulating transitory starch turnover. In our study, the temporal dynamics of 2-OG, T6P, *FBA*, and starch metabolism-related genes (*GBSS*, *AMY2*, *BMY3/BAM*) showed a “down-then-up” pattern under N deficiency, whereas G6P showed the opposite trend. This may reflect a strategic shift: initially prioritizing carbon fluxes to sustain basic energy metabolism under N deficiency, followed by enhanced carbon metabolism to compensate for energy shortages caused by N limitation (Yuan et al., 2007). These molecular events align with physiological data showing that total sugar content declined significantly at day 7 under N deficiency but was not significantly different from the control at days 14 and 21 (**Figure 2F**). Together, these findings indicate that *Z. latifolia* coordinates N assimilation and the allocation of carbon and energy not only through transcriptional regulation but also via dynamic modulation of key metabolic intermediates. This fine-tuned regulation may help maintain functions required for gall development under nutrient-limited conditions.

### 4.2 N deficiency reshapes phytohormone homeostasis and signaling during gall development

Plant hormones play pivotal roles in growth regulation and environmental response (Zhang et al., 2023b). Previous studies have shown that N supply influences plant development by modulating hormone biosynthesis, transport, and signal transduction (Krouk et al., 2011; Araújo et al., 2014; Waadt et al., 2022; Xing et al., 2023). Our multi-omics analysis revealed extensive reprogramming of hormone biosynthesis and signaling pathways in response to N deficiency (**Figures 5D, F**), suggesting a potential coordination between gall development and stress adaptation. In this study, indole-3-acetic acid (IAA) biosynthesis increased in the gall, which might be predominantly derived from tryptophan metabolism, particularly via intermediates such as indole-3-pyruvic acid (IPyA) and indole-3-acetaldehyde (IAAId) (Chung and Tzeng, 2004). While YUCCA family genes are central to IAA production, the specific YUCCA member we identified differed from those previously reported in *Z. latifolia* (**Figure 5D**) (Wang et al., 2017; Zhang et al., 2021), suggesting that distinct YUCCA isoforms may function at specific developmental stages or tissues. Interestingly, the result for IAA from metabolomics was inconsistent with the targeted LC measurement (**Figures 2M**, **5F**). Given that IAA exists in both free and conjugated forms and that untargeted LC-MS provides putative rather than unambiguous discrimination of such forms, we interpreted the metabolomic IAA signal cautiously and used the targeted LC-MS result as the primary estimate of free IAA content (Korasick et al., 2013; Giebelhaus et al., 2024). This limitation may also apply to other metabolites present in multiple chemical forms, although it does not alter our pathway-level conclusions. Possibly, the coordinated decline of IAA and its related indole derivative tryptophol implies that N deficiency restricts tryptophan-derived indole fluxes, thereby limiting the synthesis and supply of bioactive auxin (**Figure 5F**). At the signaling level, transcription factors such as *SAUR39* and *ARFs*, previously linked to the response of *Z. latifolia* to *U. esculenta* infection (Li et al., 2021), were also implicated in regulating gall development under N stress. In *Arabidopsis*, approximately two-thirds of *SAUR* genes respond to auxin response factors, mediating tissue- and function-specific auxin responses (van Mourik et al., 2017). In our study, *AUX/IAA* genes were upregulated, and *ARF* gene was downregulated by N deficiency, which is consistent with impaired auxin signaling (Jung et al., 2015; Bao et al., 2024; Cohen and Strader, 2024). This was accompanied by reduced *LAX* expression and downregulation of *SAUR* genes during later stages of gall development, implying that auxin transport and cell expansion were suppressed in the developing gall (van Mourik et al., 2017; Cohen and Strader, 2024). By 14 days, the transiently elevated IAA levels induced *GH3* expression, which catalyzes the conversion of free IAA into inactive forms such as OxIAA or conjugated IAA (Bao et al., 2024), thereby contributing to the sharp decline in IAA content observed between 14 and 21 days (**Figures 2M**, **5D**). Our above results indicate that N deficiency initiates a brief rise in *YUCCA*-mediated IPyA flux that may promote auxin production during the early phase of stress. As N limitation persists, the availability of tryptophan-derived precursors progressively declines, followed by a gradual shift toward increased conjugation and degradation results in a decrease in auxin levels.

The restriction of gall growth was further exacerbated by the dysregulation of gibberellin, cytokinin, and brassinosteroid pathways. Previous research indicated that endogenous IAA, CK, and GA_3_ levels peak during gall expansion in *Z. latifolia* (Li et al., 2021). N supply regulates bioactive gibberellin (GA) levels by modulating biosynthetic and catabolic genes, such as *ZmKSs*, *ZmGA20oxs*, and *ZmGA2oxs* (Wang et al., 2020). In this study, downregulation of *GA2OX1* likely altered GA activity (**Figure 5D**). Additionally, the suppressed expression of *CKXs* genes led to cytokinin accumulation, yet downregulation of response regulators (*RRs*) impaired cytokinin signal transduction (Yeh et al., 2015; Nguyen et al., 2016), indicating that high CK levels alone were insufficient to drive the initiation of cell division and subsequent gall development. Consistent with the importance of host hormones in tumor or gall development, evidence from other smut-host systems supports that host phytohormone status is an important contributor to gall formation. For example, maize mutants with impaired gibberellin biosynthesis or signaling exhibit reduced capacities to develop *U. maydis*-induced tumors, indicating that an intact GA pathway is required for tumor growth competence (Walbot and Skibbe, 2010). In addition, *U. maydis* candidate secreted effector UMAG_02297 is also associated with targeted regulation of auxin transport and auxin-responsive genes (Schurack et al., 2021). These studies suggest that smut-induced tumor development depends not only on pathogen infection but also on the host’s ability to establish and maintain a permissive hormonal signaling environment. Furthermore, correlation network analysis (**Figure 5G**) highlighted the role of BR signaling. In our study, BR signaling was transiently activated at 7 days under N deficiency but subsequently suppressed (**Figure 5D**). This shift in BR signaling may enhance auxin polar transport, thereby promoting root elongation and expanding N acquisition zones (Anwar et al., 2019; Wang et al., 2019; Zhang et al., 2023a). Given the demonstrated roles of BR pathway genes in crop improvement (Yang et al., 2024), further functional studies are warranted to elucidate how BR signaling contributes to gall development and adaptive growth regulation in *Z. latifolia*.

Overall, these results indicate that dynamic and coordinated modulation of multiple hormone pathways is closely associated with gall size. In N deficiency, attenuation of growth-promoting hormonal signaling constrains the developmental competence of gall tissues, consistent with observations from both maize and *Z. latifolia* smut interactions where hormonal status critically shapes tumor development and expansion (Reineke et al., 2008; Bruce et al., 2011; Li et al., 2021; Zhang et al., 2021). These findings provide a mechanistic basis for the reduced gall size and restricted radial growth observed under N limitation.

### 4.3 N deficiency remodels cell wall dynamics in association with redox and defense pathway reprogramming during gall development

Under N-deficient conditions, the overproduction of reactive oxygen species (ROS) disturbs redox equilibrium, initiating a cascade of antioxidant responses (Tewari et al., 2004; Mittler, 2017). In our study, genes such as *GME* and *VTC*, along with the metabolites oxidized glutathione and reduced glutathione, were significantly downregulated (**Figure 5B**), indicating a collapse of the ascorbate–glutathione (AsA–GSH) cycle. Meanwhile, *CAT* expression was upregulated, likely as a compensatory mechanism to maintain basal H_2_O_2_ detoxification (Mittler, 2017). This capacity may be further supported by the concurrent changes in ROS-related signaling and the activation of MAPK signaling (**Figure 5B**). This response may interact with JA and ABA biosynthesis, promoting downstream lignification and polymerization processes (Liu et al., 2021; Peng et al., 2024) since we observed a marked increase in lignin and fiber content under N deficiency (**Figure 2**). This is consistent with the notion that excessive ROS contributes to cell wall hardening under N stress (Shao et al., 2020; Tarkowski et al., 2023).

The growth of plant cells depends on a fine balance between turgor-driven cell expansion and the extensibility of the cell wall, which is mediated by the reorganization of cellulose microfibrils (Jewaria et al., 2021). In this study, during gall enlargement, genes encoding cell wall-loosening proteins, such as *XTHs*, *FLAs*, *EXPs*, and *EGs*, are typically upregulated, which may facilitate rapid cell expansion (Li et al., 2021). However, under N deficiency, the expression of most of these genes was suppressed (**Figure 5E**), with the exception of *XTH3* and *XTH4*. This exception suggests a potential compensatory role for these two *XTHs* in maintaining cell wall remodeling under N stress, and they may represent candidate regulators associated with gall expansion under N-limiting conditions.

## 5 Conclusion

N deficiency significantly inhibited plant growth and photosynthetic performance in *Z. latifolia*, and constrained gall expansion by affecting hormone levels, C and N allocation, and cell wall remodeling. Meanwhile, N deficiency reduced the accumulation of proteins, amino acids, and soluble sugars, but promoting lignin and crude fiber deposition. Integrated transcriptomic and metabolomic analyses further indicated that N deficiency elicits coordinated metabolic and regulatory shifts during gall development, involving hormone signaling, stress responses, carbon metabolism, and phenylpropanoid biosynthesis. Overall, N limitation perturbs growth promotion and energy metabolism but enhances stress-responsive cell wall strengthening. These findings provide mechanistic insights into how N availability modulates developmental and metabolic processes in *Z. latifolia* and establish a molecular regulatory framework for optimizing N nutrient utilization to achieve high yield and quality in its cultivation.

## Data Availability

The original contributions presented in the study are included in the article/**Supplementary Material**. Further inquiries can be directed to the corresponding authors.
