# Supplementary material for: Nitrogen deficiency restricts gall development by altering metabolic and hormonal networks in Zizania latifolia
Source: Front Plant Sci. 2026 May 7;17:1754728. doi: 10.3389/fpls.2026.1754728 (PMC13190173; doi:10.3389/fpls.2026.1754728)
Supplement: Supplementary file 1 [file DataSheet1.pdf]

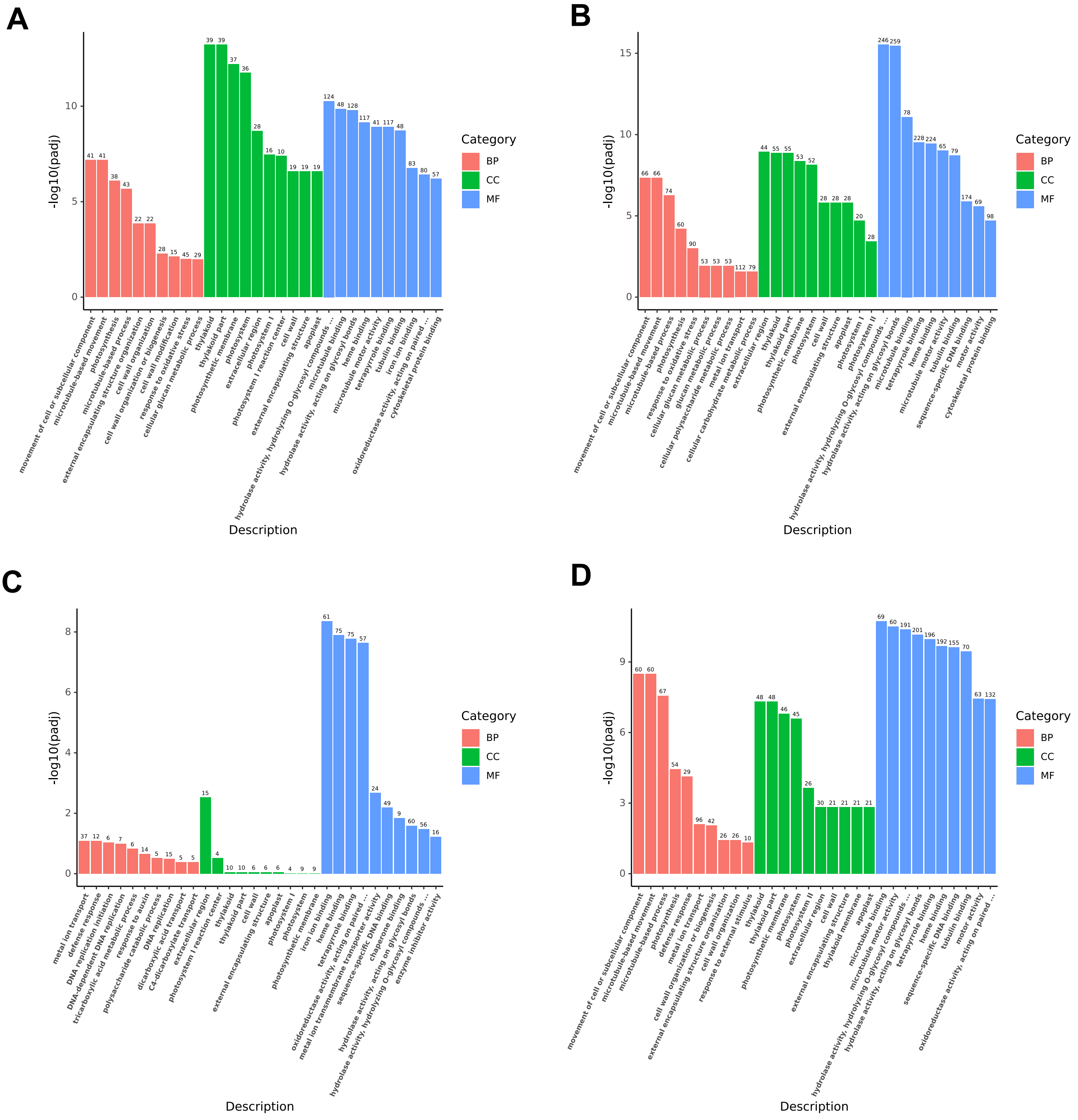
A
-log10(padj)
0
5
10
41 41 38 43 22 22 28 15 45 29 39 39 37 36 28 16 10 19 19 19 124 48 128 117 41 117 48 83 80 57
movement of cell or subcellular component
microtubule-based movement
photosynthesis
microtubule-based process
external encapsulating structure organization
cell wall organization
cell wall organization or biogenesis
cell wall modification
response to oxidative stress
cellular glucan metabolic process
thylakoid
thylakoid part
photosynthetic membrane
photosystem
extracellular region
photosystem I
photosystem I reaction center
cell wall
external encapsulating structure
apoplast
hydrolase activity, hydrolyzing O-glycosyl compounds ...
microtubule binding
hydrolase activity, acting on glycosyl bonds
heme binding
microtubule motor activity
tetrapyrrole binding
tubulin binding
iron ion binding
oxidoreductase activity, acting on paired ...
cytoskeletal protein binding
Description
Category
BP
CC
MF
B
-log10(padj)
0
5
10
15
66 66 74 60 90 53 53 53 112 79 44 55 55 53 52 28 28 28 20 28 246 259 78 228 224 65 79 174 69 98
movement of cell or subcellular component
microtubule-based movement
microtubule-based process
photosynthesis
response to oxidative stress
cellular glucan metabolic process
glucan metabolic process
cellular polysaccharide metabolic process
metal ion transport
cellular carbohydrate metabolic process
extracellular region
thylakoid
thylakoid part
photosynthetic membrane
photosystem
cell wall
external encapsulating structure
apoplast
photosystem I
photosystem II
hydrolase activity, hydrolyzing O-glycosyl compounds ...
hydrolase activity, acting on glycosyl bonds
microtubule binding
tetrapyrrole binding
heme binding
microtubule motor activity
tubulin binding
sequence-specific DNA binding
motor activity
cytoskeletal protein binding
Description
Category
BP
CC
MF
C
-log10(padj)
0
2
4
6
8
37 12 6 7 6 14 5 15 5 5 15 4 10 10 6 6 6 4 9 9 61 75 75 57 24 49 9 60 56 16
metal ion transport
defense response
DNA replication initiation
DNA-dependent DNA replication
tricarboxylic acid metabolic process
response to auxin
polysaccharide catabolic process
DNA replication
dicarboxylic acid transport
C4-dicarboxylate transport
extracellular region
photosystem I reaction center
thylakoid
thylakoid part
cell wall
external encapsulating structure
apoplast
photosystem I
photosystem
photosynthetic membrane
iron ion binding
heme binding
tetrapyrrole binding
oxidoreductase activity, acting on paired ...
metal ion transmembrane transporter activity
sequence-specific DNA binding
chaperone binding
hydrolase activity, acting on glycosyl bonds
hydrolase activity, hydrolyzing O-glycosyl compounds ...
enzyme inhibitor activity
Description
Category
BP
CC
MF
D
-log10(padj)
0
3
6
9
60 60 67 54 29 96 42 26 26 10 48 48 46 45 26 30 21 21 21 21 69 60 191 201 196 192 155 70 63 132
movement of cell or subcellular component
microtubule-based movement
microtubule-based process
photosynthesis
defense response
metal ion transport
cell wall organization or biogenesis
external encapsulating structure organization
cell wall organization
response to external stimulus
thylakoid
thylakoid part
photosynthetic membrane
photosystem
photosystem II
extracellular region
cell wall
external encapsulating structure
thylakoid membrane
apoplast
microtubule binding
microtubule motor activity
hydrolase activity, hydrolyzing O-glycosyl compounds ...
hydrolase activity, acting on glycosyl bonds
tetrapyrrole binding
heme binding
sequence-specific DNA binding
tubulin binding
motor activity
oxidoreductase activity, acting on paired ...
Description
Category
BP
CC
MF

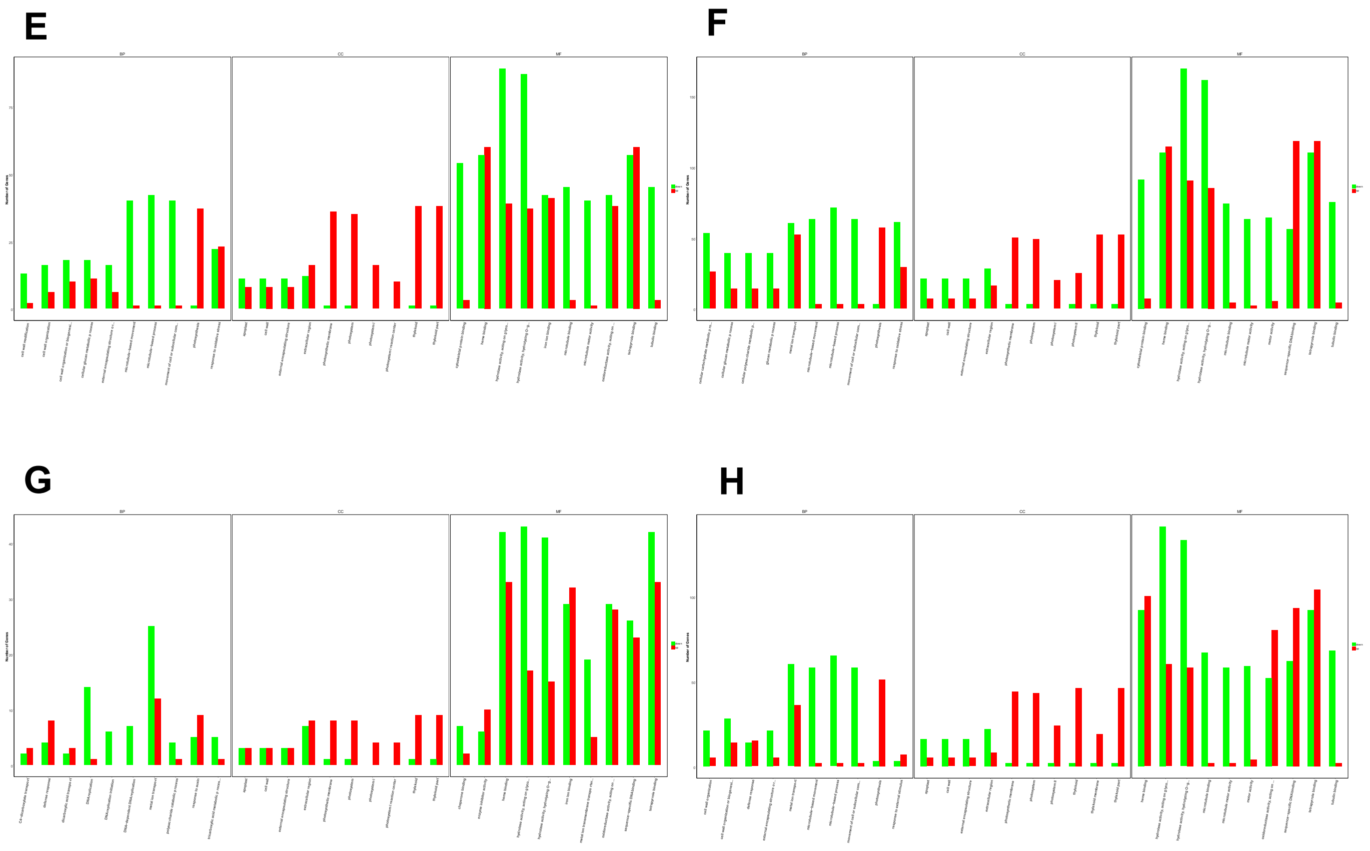

Figure S1. GO enrichment analysis of differentially expressed genes.
(A.E) CK_14_vs_CK_7. (B.F) DN_14_vs_DN_7. (C.G) DN_7_vs_CK_7. (D.H) DN_14_vs_CK_14.

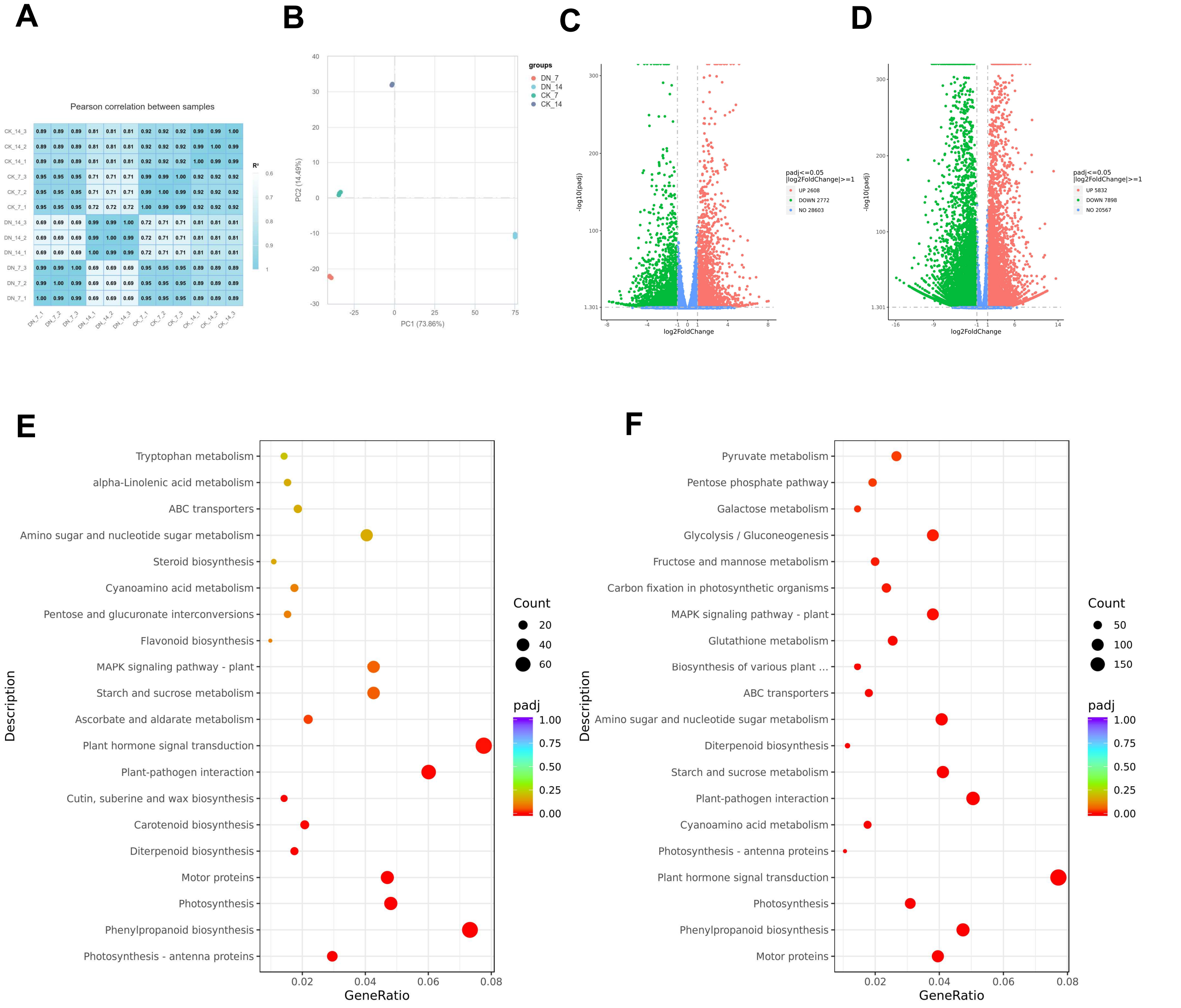

Figure S2. Transcriptomic landscape of N-deficient galls.

(A) Pearson correlation heatmap. (B) PCA plot (PC1 vs PC2). (C, D) Volcano plots for (C) CK_14 vs CK_7, (D) DN_14 vs DN_7 (red: up; green: down; blue: NS; $|\log_2 FC| \geq 1$, FDR<0.05). (E-F) KEGG enrichment for (E) CK_14 vs CK_7, (F) DN_14 vs DN_7.

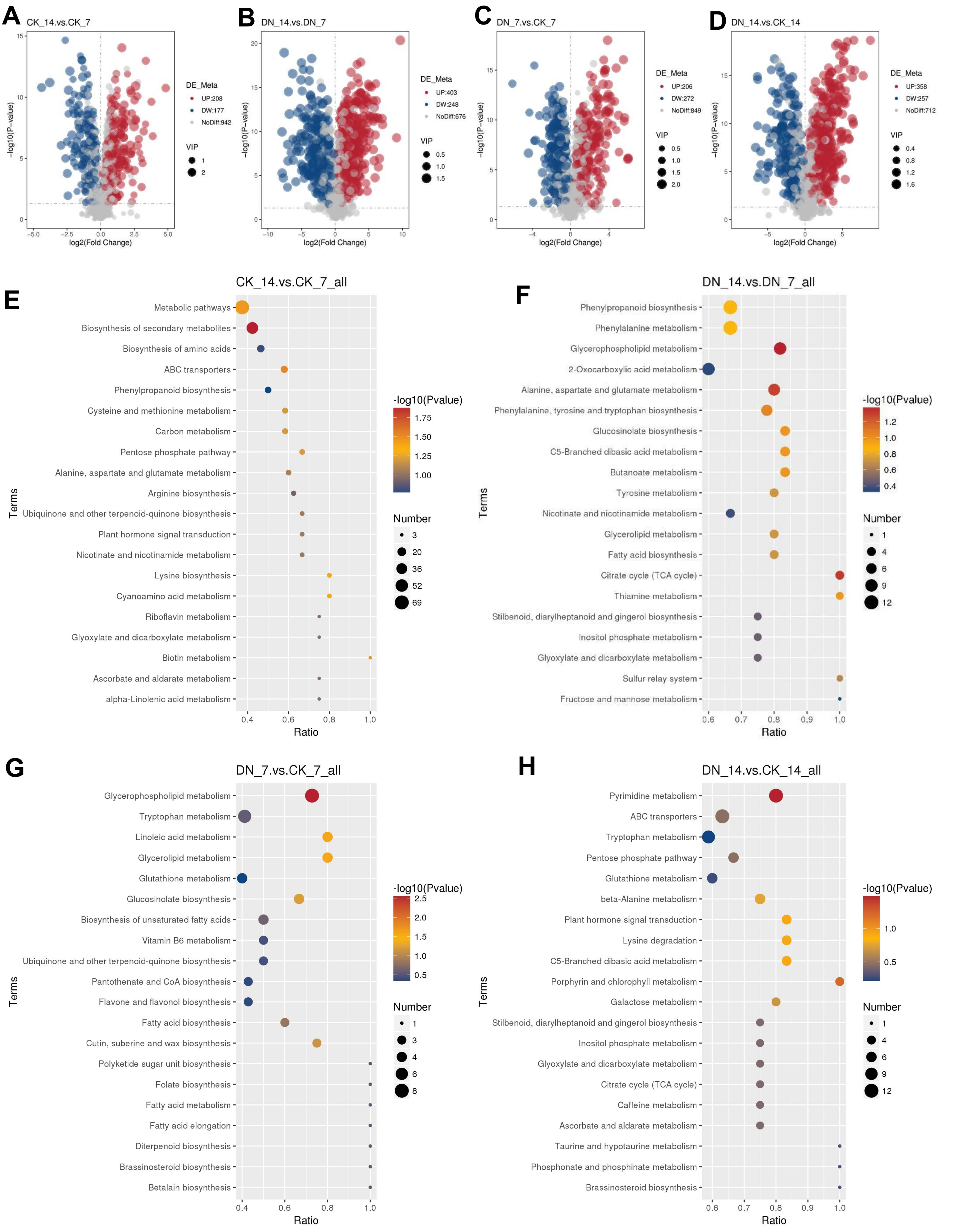

Figure S3. The volcano graph and kegg enrich scatterplot of differentially accumulated metabolites (DAMs).

(A.E) CK_14_vs_CK_7. (B.F) DN_14_vs_DN_7. (C.G) DN_7_vs_CK_7. (D.H) DN_14_vs_CK_14. Red: up; blue, down.

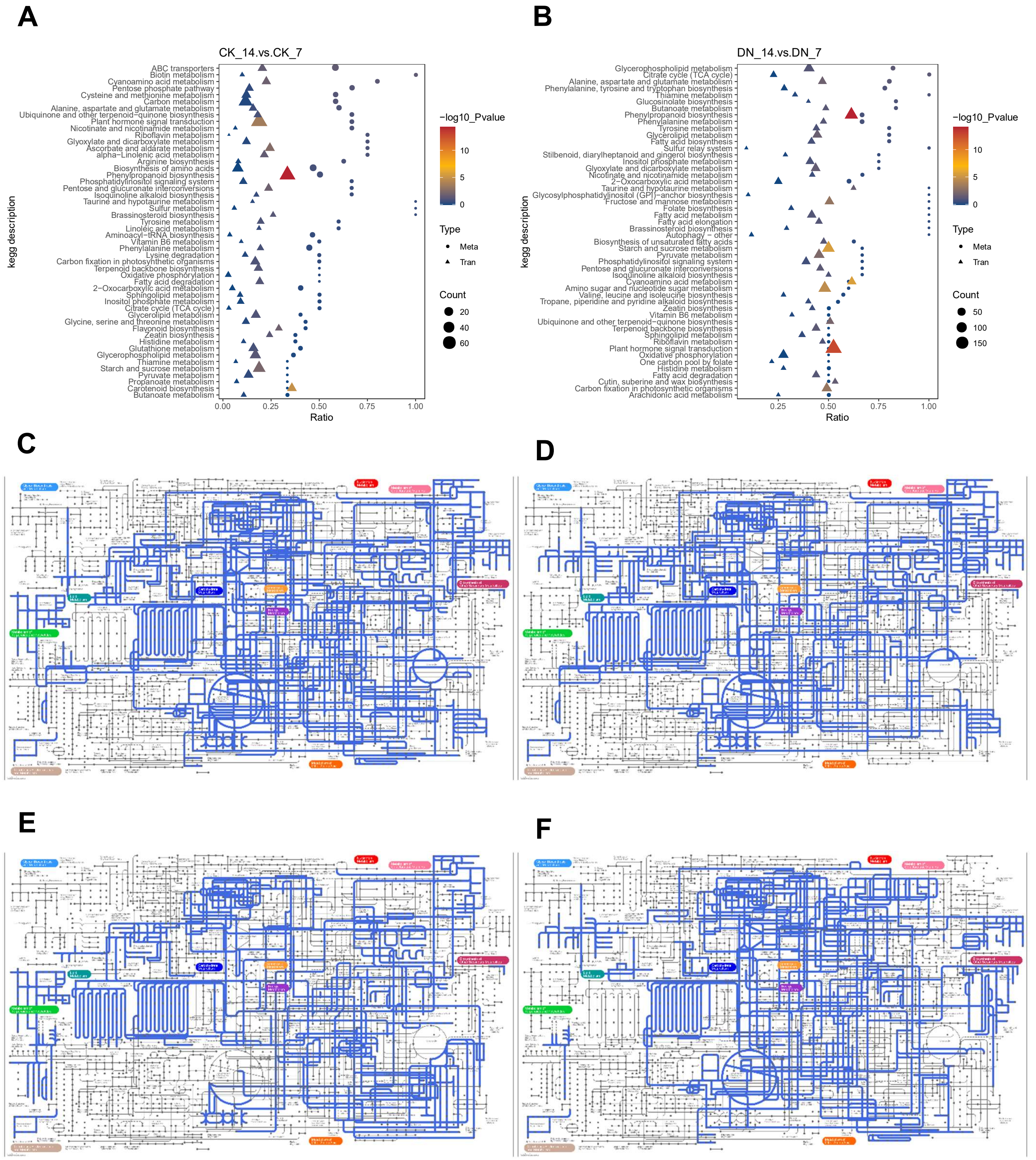

Figure S4. Pathway association analysis of metabolic-transcriptional corresponding comparison pairs. (A, C) CK_14_vs_CK_7. (B, D) DN_14_vs_DN_7. (E) DN_7_vs_CK_7. (F) DN_14_vs_CK_14. The bold-colored lines indicate common pathways enriched by the two omics.
